# Colorimetric Detection of Chromium(VI) Ions in Water Using Unfolded-Fullerene Carbon Nanoparticles

**DOI:** 10.3390/s21196353

**Published:** 2021-09-23

**Authors:** Saeedeh Babazadeh, Ramanand Bisauriya, Marilena Carbone, Ludovica Roselli, Daniele Cecchetti, Elvira Maria Bauer, Simona Sennato, Paolo Prosposito, Roberto Pizzoferrato

**Affiliations:** 1 Department of Industrial Engineering, University of Rome Tor Vergata, 00133 Rome, Italy; s.babazadeh1990@gmail.com (S.B.); r.bisauriya@gmail.com (R.B.); paolo.prosposito@uniroma2.it (P.P.); 2 Department of Mechanical Engineering of Biosystems, Agriculture Faculty, Urmia University, Urmia 5756151818, Iran; 3 Department of Chemical Science and Technologies, University of Rome Tor Vergata, 00133 Rome, Italy; ludovica.roselli@alumni.uniroma2.eu (L.R.); daniele.cecchetti@uniroma2.it (D.C.); 4 Institute of Structure of Matter (ISM), Italian National Research Council (CNR), 00015 Rome, Italy; elvira.bauer@ism.cnr.it; 5 Institute for Complex Systems (ISC), Italian National Research Council (CNR) and Physics Department, Sapienza University of Rome, 00185 Rome, Italy; simona.sennato@roma1.infn.it

**Keywords:** optical sensing, water quality, hexavalent chromium, colorimetry, carbon nanomaterials

## Abstract

Water pollution caused by hexavalent chromium (Cr(VI)) ions represents a serious hazard for human health due to the high systemic toxicity and carcinogenic nature of this metal species. The optical sensing of Cr(VI) through specifically engineered nanomaterials has recently emerged as a versatile strategy for the application to easy-to-use and cheap monitoring devices. In this study, a one-pot oxidative method was developed for the cage opening of C_60_ fullerene and the synthesis of stable suspensions of N-doped carbon dots in water–THF solutions (N-CDs-W-THF). The N-CDs-W-THF selectively showed variations of optical absorbance in the presence of Cr(VI) ions in water through the arising of a distinct absorption band peaking at 550 nm, i.e., in the transparency region of pristine material. Absorbance increased linearly, with the ion concentration in the range 1–100 µM, thus enabling visual and ratiometric determination with a limit of detection (LOD) of 300 nM. Selectivity and possible interference effects were tested over the 11 other most common heavy metal ions. The sensing process occurred without the need for any other reactant or treatment at neutral pH and within 1 min after the addition of chromium ions, both in deionized and in real water samples.

## 1. Introduction

The pollution of drinking water due to heavy metals (HMs) represents a serious hazard for human health since increasing industrialization, illegal or poor waste dumping practices and, for some metal species, natural occurrence have become worldwide issues. Despite a growing public concern followed by increasingly severe legislative actions, a significant part of the world population still has to deal with high doses of HMs, as chronic poisoning has been deemed to be a real fact for at least one million people in developing countries [1]. Moreover, HMs in surface waters and seawater affect human health indirectly through food chain accumulation. The toxicity of HMs depends on several factors including the chemical species, dose, and route of exposure. Hexavalent chromium (Cr(VI)) is one of the most common water contaminants since it comes from a variety of industrial processes, such as chrome-plating, leather tanning, printing, and pigment production. It is also considered very harmful since it can cause damage to multiple organs, even at low concentrations, and it has been classified as a human carcinogen [2]. For this reason, the Guidelines for Drinking-Water Quality by the World Health Organization (WHO) [3] have set the Cr(VI) concentration limits in the ppb range, e.g., 50 µg/L, corresponding to a molar concentration of 0.960 µM.

The determination and quantification of hexavalent chromium ions are generally performed with well-established laboratory methods such as atomic absorption spectrophotometry (AAS), high performance liquid chromatography (HPLC), inductively coupled plasma mass spectrometry (ICP-MS), flame atomic absorption spectroscopy (FAAS), atomic fluorescence spectroscopy (AFS), and graphite furnace atomic spectroscopy (GFAAS) [4,5,6]. While these techniques provide an accurate trace-level reliable evaluation of concentration, they require bulky, heavy, and expensive instrumentation; time consuming procedures; and highly skilled staff. These factors obviously limit the mass control of water quality, especially in vast and remote areas. Within this framework, much attention has been devoted to the development of simple, fast, portable, and cost-effective monitoring methods that can be used onsite by the broadest possible range of operators.

Optical sensing has recently been demonstrated to be a possible answer to such a great demand for friendly and affordable methods for Cr(VI) monitoring. Based on optical colorimetric and/or fluorometric responses, it is possible to envisage applications of cheap and disposable paper-based detection, miniaturized lab-on-chip (LOC) technology and cost-effective handheld smartphone-based devices [7]. Moreover, optical sensing is easily feasible in water assays, the natural media for chromium contamination and the base for microfluidic systems, which allow sampling on a sub-milliliter scale [8]. For this reason, extensive research has been conducted to develop specific reactants and dyes that change their absorbance or fluorescence emission in the presence of Cr(VI) in water. For instance, Lace et al. have recently optimized a colorimetric method based on 1,5-diphenylcarbazide with a limit of detection (LOD) of 0.46 µM [9].

The exploitation of sensing nanomaterials, with peculiar tunable optical properties and a large interaction area, has provided further stimulus to this field of research towards a huge number of biological and chemical analytes, from gases to HM ions [10]. By exploiting the colorimetric response associated with the surface plasmon resonance (SPR) in gold nanorods, Li’s group has demonstrated the colorimetric detection of Cr(VI) in drinking and sea water with a LOD as low as 88 nM [11]. Liu et al. [12] obtained a LOD of 300 nM for Cr(III) and Cr(VI) through citrate-capped Au nanoparticles. By using polyvinylpyrrolidone (PVP) functionalized silver nanoparticles, He et al. achieved the very low LOD value of 34 nM for Cr(VI) [13]. However, the synthesis of metal nanoparticles and the capping procedures do not allow for the simple control of size, which reflects the reproducibility of the optical properties of the sensing medium. Carbon-based nanoparticles have recently been investigated for their sensitivity to HMs trough fluorescence quenching [14,15]. Graphene-oxide nanoparticles have demonstrated sensitivity to Cr(VI) with a remarkably low LOD of 6 nM through a peroxidase mimetic catalyst mechanism [16]. However, this imposing result required quite a complicated procedure, including the mixing of four different compounds in a phosphate-buffered saline solution in order to maintain a pH of 3.5 immediately before the sensing experiment and a 30-min incubation period at room temperature once the contaminated assay was added. Moreover, the linearity of the response was limited to a very small range of 0.43 μM.

Our group has recently investigated the sensitivity of fluorescent carbon nanoparticle water suspensions synthesized by means of cage-opening C_60_ fullerene via a modified Hummers’ reaction [17,18]. By measuring the simultaneous changes of optical absorbance and fluorescence intensity, we observed the proof of concept of the multiple and selective sensing of copper, lead, arsenic, and cadmium in the same water solution. Absorbance variations, however, occurred in the UV spectral range, which is not easily measurable.

In this study, we used a different synthetic approach, where fullerene opening was conducted by a hydroxyl radical reaction in basic ambient. This allowed the insertion of the targeted functional groups on the unfolded fullerene as well as a size control. Selectivity towards Cr(VI) and the associated signal enhancement were achieved by means of the employment of a post-synthesis addition of THF. In this novel approach, hydrogen bonds are created between the functional groups on the surface of the quantum dots and the THF, which may help to somewhat decrease the size of the aggregates [19] and to selectively expose Cr(VI)-sensitive functional groups. Using this method, we obtained N-doped carbon dots in water and THF (N-CDs-W-THF) that selectively showed absorbance variations in the visible range, i.e., from 500 to 650 nm, in the presence of Cr(VI) ions in water. Since that is the transparency region of the N-CDs-W-THF solution and since the absorbance increases linearly with the ion concentration, a straightforward chromium concentration determination that is simply based on a ratiometric evaluation can be accomplished. The sensing solutions can be prepared in advance and can be stored for a long time due to a demonstrated 9-month stability, while the measurements can be accomplished in 1 min with the simple addition of water samples without the need for any other reactant, treatment, or pH buffer.

## 2. Materials and Methods

### 2.1. Materials

C_60_ buckminsterfullerene, hydrogen peroxide solution (30%), and THF were purchased from Sigma–Aldrich (St. Louis, MO, USA). Ammonium hydroxide solution (28%) was ordered from VWR Chemicals (VWR International, Radnor, PA, USA). The water used in all of the experiments was doubly distilled and purified using a Milli-Q system (Millipore, Milford, MA, USA). All of the metal salts (AgNO_3_, Cd(NO_3_)_2_, Cu(NO_3_)_2_, CrCl_3_, Na_3_AsO_3_, CoCl_2_, FeCl_2_, FeCl_3_, HgCl_2_, NiCl_2_, PbCl_2_, K_2_Cr2O_7_, CaCl_2_) were purchased from Merck Chemicals. All of the chemicals used in this work were of analytical grade. The metal salt solutions to be used for sensitivity measurements were prepared at the concentration of 1000 µM in deionized water the day before use and were stored in the dark at 5 °C to avoid degradation due to accidental collateral reactions. The stock salt solutions were then diluted to the appropriate concentration immediately before the sensitivity measurements.

### 2.2. Synthesis of N-CDs

The oxidative opening and doping of fullerene and functionalization was performed by dissolving 40 mg of fullerene in 200 mL deionised water and sonicating it for 1 h. The slurry was subsequently transferred to a round bottom flask with the addition of 160 mL of H_2_O_2_ (30%) and 40 mL of NH_4_OH (28%) dropwise while stirring and was heated in an oil bath at 110 °C for 3 h under reflux [20]. The resulting products were centrifuged at 3500 rpm for 50 min. The ensuing supernatant was gently heated at 40 °C overnight to remove residual H_2_O_2_ (visual de-bubbling). The post-synthesis addition of THF was achieved by mixing de-bubbled supernatant and THF in a ratio 1:1 and by centrifuging it at 3500 rpm until the disappearance of the residues on the bottom of the test tube (minimum 200 min). The resulting solution was diluted with water and THF in the ratio 1:1 to obtain the final sensing solution with a N-CD concentration of approximately 100 µM.

### 2.3. Instrumentation for Characterization and Sensitivity Measurements

Infrared spectra were recorded with a Shimadzu Prestige-21 FT-IR instrument equipped with an attenuated total reflectance (ATR) diamond crystal (Specac Golden Gate) in the range of 400–4000 cm^−1^ with a resolution of 4 cm^−1^. A layer of N-CDs was deposited by drop casting on a clean Al foil used as a sample holder and were oven dried before measurement. Determination of hydrodynamic size and size distribution of pristine N-CDs and upon interaction with Cr(VI) was conducted by dynamic light scattering (DLS) with a Malvern NanoZetaSizer apparatus (Malvern Instruments LTD, Malvern, UK) equipped with a 5 mW HeNe laser and temperature controlled with a Peltier system and backscattering detection. This configuration is less sensitive to multiple scattering effects and dust than 90° geometry. The outcomes were analysed by means of the NNLS algorithm. SEMs images were collected with a Zeiss Auriga Field Emission-Scanning Electron Microscope instrument operating at 7 kV. Fluorescence spectra were recorded by using a laboratory set-up for photoluminescence (PL) measurements with a spectral bandpass of 3 nm for the emitted light [21]. The spectral response of the set up was calibrated with a reference Spectral Fluorescence Standards Kit (Sigma Aldrich, Milano, Italy). This enabled the full correction of the emission curves over the range 300–700 nm. The pH of the solutions was measured with a digital pH meter (Hanna Instruments, Padova, Italy). A Cary 50 spectrophotometer (Varian Inc., Palo Alto, CA, USA) was used to record the UV–vis absorption spectra of the sample solutions for the colorimetric determination of the Cr(VI) concentration. Samples were held in fused silica cuvettes with a 10 mm optical path both for the PL and absorption measurements.

### 2.4. Procedure and Optimization for Sensitivity Measurements

All of the measurements for the colorimetric detection of the Cr(VI) ions were performed against a reference (blank) solution that was prepared by mixing 2 mL of the N-CDs-W-THF sensing solution with 1 mL of deionized water (18.25 MΩ cm). The typical measurement in the presence of metal ions was conducted by mixing 2 mL of the N-CDs-W-THF sensing solution with 1 mL of a HM salt solution in deionized water at the appropriate concentration of the metal ion. The ratio 2:1 was chosen after optimization tests for maximizing the sensitivity without adding too much water to the sensing solution. After gently mixing the solution for 1 min, the absorbance spectra were recorded. The same procedure was adopted for the ion concentration determination in real water samples, the only difference being that the salt solutions were prepared using the water as collected from the real source, without filtration or treatment. All of the water samples were tested in triplicate. Stability of the N-CDs-W-THF sensing solution was verified over a 9-month time period in the dark at 4 °C and for at least 6 months in a normally lit room at 21 °C.

## 3. Results and Discussion

### 3.1. Morphological, Structural and Optical Characterization of N-CDs

The samples were characterized by FT-IR spectroscopy to determine the type of functional groups present during synthesis and the ensuing post-synthesis addition of THF. Furthermore, the average size was estimated by DLS and SEM imaging. Subsequently, the optical properties were probed by means of absorbance as well as fluorescence spectroscopies in the UV-vis regions.

**Figure 1:**
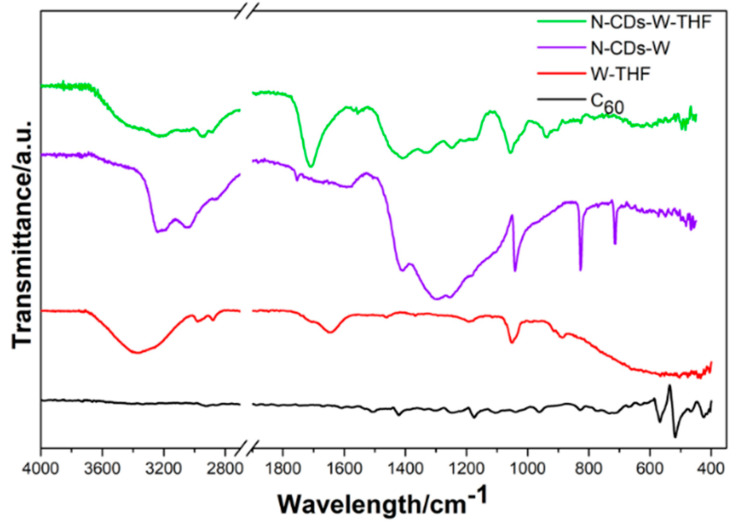
Infrared spectra of pristine fullerene-C_60_ (black solid line), mixture water-THF (red solid line), N-CDs-W (violet solid line), and N-CDs-W-THF (green solid line). The samples were deposited on clean Al foil by drop casting prior to the measurement.

The infrared spectra of N-CDs-W and N-CDs-W-THF are reported in Figure 1. The IR spectra of fullerene and of the mixture of water–THF are also reported for comparison purposes. The summary of the IR bands is reported in Table S1 in Supporting Information. The ATR-FT-IR spectra of the N-CDs-W and N-CDs-W-THF dispersions were registered after the drop-casting of the solutions on clean Al foils. After 48 h of drying, the ensuing samples were transferred onto the ATR-FT-IR crystals for direct measurements. Both the N-CDs-W and N-CDs-W-THF appear quite different with respect to the pristine fullerene powder, which is characterized by only four vibration modes located at 522 cm^−1^, 572 cm^−1^, 1180 cm^−1^, and 1427 cm^−1^ due to the radial displacements of the carbon atoms and the tangential modes of the carbon atoms [22]. In contrast, the region between 3500–2500 cm^−1^ as well as the region below 1700 cm^−1^ of the CDs samples display a large number of bands. As far as N-CDs-W is concerned, the carboxylic, amidic, hydroxylic, and amine groups are introduced upon opening. In particular, the IR spectrum shows four broad absorptions between 3500 cm^−1^ and 2500 cm^−1^ due to the overlap of the hydroxyl –OH stretching centered at 3430 cm^−1^ with the –NH stretching (3200 cm^−1^), –CH=CH– (3066 cm^−1^) aromatic stretching, and –CH bond stretching of the sp^3^ carbons (2881 cm^−1^) [23,24]. The appearance of a broad absorption around 3200 cm^−1^ clearly indicates the presence of N-based functional groups.

The presence of amine/amide functional groups differentiate the current preparation from the unfolded-fullerene carbon nanoparticles previously reported by Ciotta et al. [17,18] by using a modification of the classical Hummer method, where only the hydroxylic or carboxylic functional groups were detected. The N-CDs-W also contain carboxylic and hydroxylic groups, as ascertained by a quite weak but sharp peak centered at 1753 cm^−1^ [22]. Interestingly two very weak and broad absorbances were detected between 1700 and 1590 cm^−1^. Specifically, the shoulder located around 1683 cm^−1^ may be assigned to the C=O stretching of an amide functional group, whereas the shoulder below 1600 cm^−1^, i.e., 1598 cm^−1^, falls in the spectral region that is characteristic for –C=C– aromatic stretching, –NH_2_ scissoring, and –C=O stretching, preventing assignment [23]. The quite intense and broad peak located at 1409 cm^−1^ is probably related to the –C-N stretching of primary amides, but the –C–O stretching of the hydroxyl groups in fullerenols can also be found in the same spectral region. [22,23,24,25]. The region between 1310–1100 cm^−1^ is characterized by several large and intense peaks that fall in a region that is characteristic for –C–H bending vibrations and various C–O and C-N bond stretchings. A quite weak but evident shoulder centered around 1100 cm^−1^ may be due to the formation of ether (–C–O–C) functions [22]. The presence of hydroxyl groups is deductible from a sharp signal located at 1042 cm^−1^ and can be assigned to the –C–C–OH stretching. In addition, a sharp absorption is observed below 1000 cm^−1^ and might be related to the symmetric –C–C–O hydroxyl stretching (827 cm^−1^) or –C–O–C- bending of isolated epoxy moieties on the CDs surface [22,26]. Finally, the sharp signal observed at 714 cm^−1^ might be related to the –NH_2_ wagging motions.

The addition of THF to the aqueous solution of N-CDs resulted in significant changes in all of the spectral regions. The dried deposit of N-CDs-W-THF showed an increase of the –OH stretching absorption around 3390 cm^−1^ as well as more evident signals below 3000 cm^−1^ centered around 2953 cm^−1^ and 2882 cm^−1^, respectively, which is typical for sp^3^ –CH stretching vibrations. A broad –N–H asymmetric stretching signal was observed at 3205 cm^−1^. The absorption characteristic for –CH=CH– stretching (3053 cm^−1^) appears of larger intensities with respect to the N-CDs-W. The largest differences between N-CDs-W and N-CDs-W-THF are observed in the region characteristic for –C=O carboxylic acid stretching. A strong and broad absorption appears at 1707 cm^−1^, whereas the sharp and weak signal observed for N-CDs-W at 1753 cm^−1^ is completely absent. This behavior generally indicates strong hydrogen bonding. In the present case, this may be related to the interaction between the oxygen atom of THF and the carboxylic acid groups on the surface of the CDs. Furthermore, the N-CDs-W-THF sample shows a 20 cm^−1^ shift of the –C-OH carboxylic acid stretching absorption towards a higher wavelength (1329 cm^−1^), thus supporting the formation of strong hydrogen bonding. The primary amide C=O stretching at 1672 cm^−1^ is absent, though amide –C–N stretching can be observed at 1409 cm^−1^, but it is not uniquely assigned [24].

A broad peak centered at 1055 cm^−1^ with two shoulders on the tail of the lower wavelengths can be assigned to –C–O–C asymmetric ether stretching (which is also possibly from the THF–water mixture). Three absorptions are present at 935, 905, and 874 cm^−1^. Their assignment is also not unique since they may be related to the –NH wagging of the secondary amines as well as to hydrogen bonding interactions between THF and different hydroxyl groups. In this scenario, the intensity of the fullerene epoxy bending at 824 cm^−1^ decreases, and remarkably, the primary amine wagging vibration at 714 cm^−1^ is absent. In comparison, the THF–water mixtures are characterized by the intense –C–O–C– asymmetric stretching vibrations located at around 1055 cm^−1^ and the symmetric C–O–C stretching vibrations below 1000 cm^−1^ are affected by hydrogen bonding, mostly in regions where the samples do not have any absorptions.

In summary, the largest differences between the N-CDs-W and N-CDs-W-THF IR spectra are related to the strong hydrogen bonding between THF and the hydroxyl or carboxylic groups in the latter as well as a selection of the N-containing groups towards the secondary amines and the tertiary amides.

The average size of the synthesized N-CDs-W and N-CDs-W-THF was determined by DLS, and the outcomes are reported in Table 1, including the error. The average diameter of the N-CDs-W sample was 3.50 nm and decreased to 3.12 nm with the addition of THF and prolonged centrifugation. This trend, though apparently unexpected [19], is consistent with the strong hydrogen bonding between THF and the functional groups of the N-CDs-W-THF. More specifically, the THF addition may break down somewhat bigger aggregates, and the interaction with the surface functionalities prevents from re-aggregation.

As a result, smaller and more stable CDs-THF moieties were formed. In order to have an insight into the interaction between the N-CDs-W-THF and the metal ions, a DLS measurement was conducted upon mixing N-CDs-W-THF with a water solution containing 5 mM of Cr(VI). The resulting average diameter was 4.00 nm, thus larger than the radius of the CDs alone and compatible with the addition of Cr(VI) (ionic radius in vacuum 0.128 nm) and its own solvation shell.

**Figure 2:**
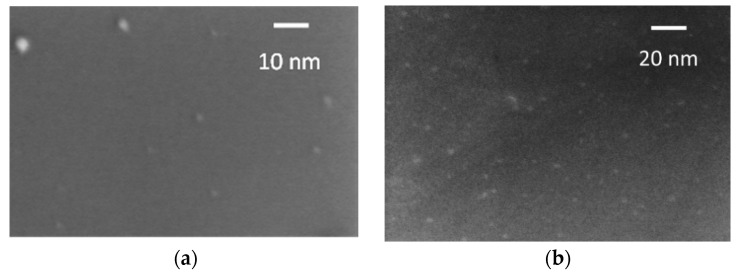
(**a**) SEM image of the N-CDs-W sample; (**b**) SEM image of the N-CDs-W-THF sample.

The SEM images of the very diluted solutions (1:100) also indicate the presence of particles in the 3 nm range (Figure 2). The X-ray diffraction pattern of the synthesized N-CDs is reported in Figure S1 of the Supplementary Materials, which was formed upon solvent evaporation and deposition on an Al sample-holder. It is characterized by a broad band centered at 2*θ* = 25°, similar to the CDs obtained by bottom-up approaches, such as pyrolysis or other high-temperature methods. The natural broadening is attributed to the small size of the domains and turbostratic disorders [27,28]. The presence of the functional groups on the CDs also plays a role in preventing the ordering of the dots and the narrowing of the diffraction peak. Additional crystalline features are observed in the 2*θ* range at 18°–35°, likely related to a graphitization process during the solvent evaporation (performed at 150 °C) for measurement purposes and unreacted fullerene (JCPDS 43-995) (which do not contribute to the UV-Vis spectra nor to the sensing process anyway).

**Figure 3:**
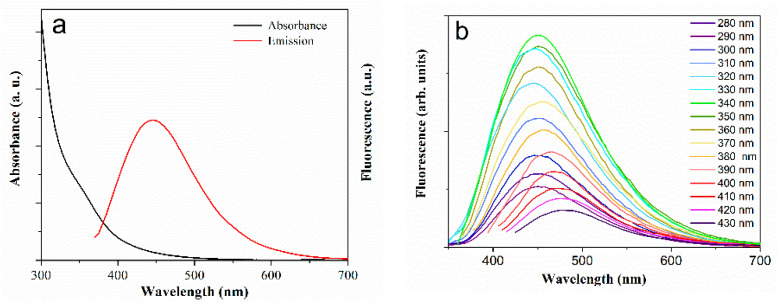
(**a**) UV-vis absorption spectrum (black curve) and fluorescence spectrum (red) of N-CDs-W-THF solution excited at λ_exc_ = 355 nm; (**b**) emission spectra at different excitation wavelengths.

Figure 3a displays the absorbance and fluorescence spectra of the N-CDs-W-THF solution. The increasing absorbance with decreasing wavelength is due to the tail of the high-energy optical π-π* transitions of the isolated sp^2^-carbon domains in the graphene-like defective lattice. A very smooth shoulder can be seen at ∼355 nm and is generally attributed to the n-π* transitions of oxygen-containing and nitrogen-containing functional groups, such as the hydroxyl, carboxyl, epoxy moieties, and primary amides that are located both in the basal plane and at the edge of the carbon structure. We note that a similar absorption structure was also observed in the material prepared with the original method [17], where it was shifted by approximately 50 nm towards the UV range. Figure 3b and Figure S2 show the slightly excitation-dependent nature of the fluorescent emission, as often reported in the literature [14,15]. In particular, the excitation peak at approximately 350 nm is consistent with the “antenna” role of the smooth shoulder at 355 nm in the absorption spectrum.

### 3.2. Colorimetric Sensing of Cr(VI) Ions

**Figure 4:**
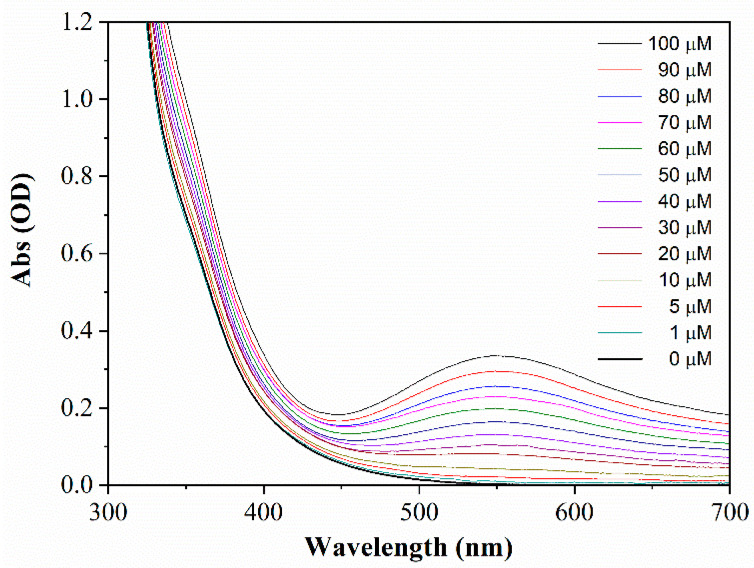
UV-vis absorption spectra of N-CDs-W-THF upon the addition of Cr(VI) ions at different concentrations.

**Scheme 1:**
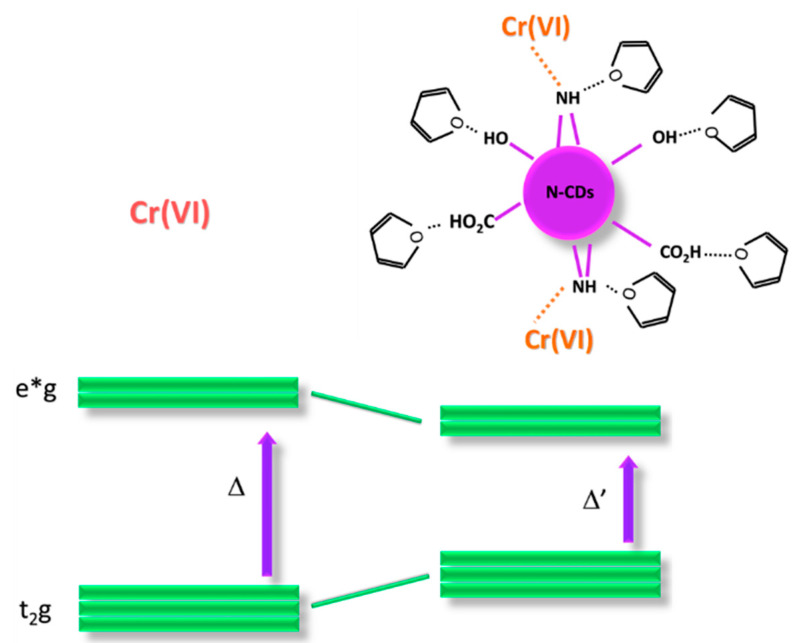
Cr(VI) and the corresponding energy gap (Δ), which corresponds to the transition energy, right side, selective interaction of Cr(VI) with N-CDs hydrogen bonded with THF, and corresponding reduction of the energy gap (Δ’).

Figure 4 shows the UV-vis absorption spectra of the N-CDs-W-THF sensing solution after the addition of different concentrations of Cr(VI). As it can be observed, the presence of chromium ions produced the arising of a distinct absorption band peak at 550 nm, with increasing intensity as the ion concentration increased. The change in absorbance became visually clear at concentrations above 30 µM, with the assay solution turning from a clear transparency to a blue-violet color (Figure S3). Interestingly, the arising of the band in the visible range parallels the strong decrease of the typical absorption band of the chromium ions at 355 nm, which becomes a very smooth shoulder in the absorption spectrum of N-CDs-W-THF after the addition of Cr(VI) (Figure S4). This suggests that the interaction of the metal ion with the carbon nanoparticle is quite strong, possibly forming a complex so as to alter the external electronic levels of chromium and to produce new optical transitions in the visible range, as shown in Scheme 1. On the other hand, the role of the post-synthesis addition of THF is evidenced by the fact that the N-CDs-W did not show any response around 550 nm upon the addition of Cr(VI), as reported in Figure S4 (green curve).

**Figure 5:**
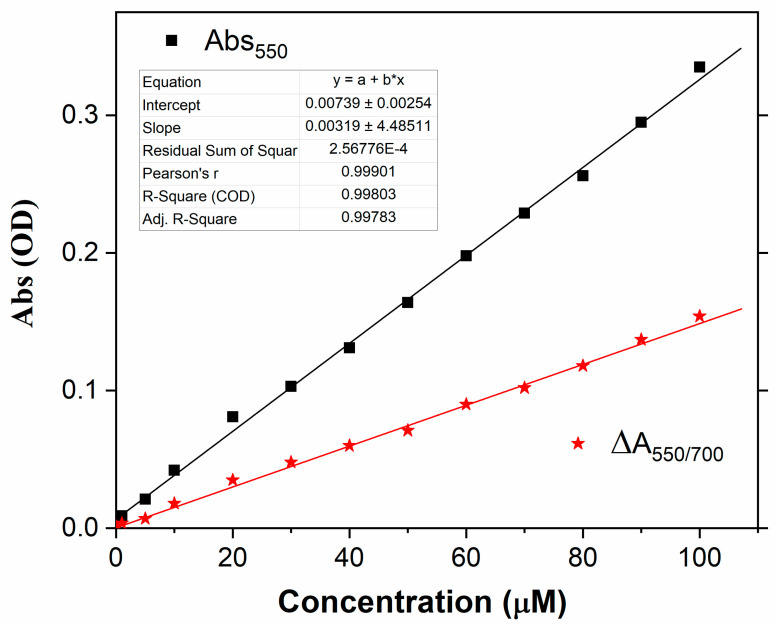
Absorbance at 550 nm (squares) and absorbance difference ΔA_550/700_ as a function of Cr(VI) concentration in water.

The relationship between the increment of absorbance at 550 nm and the Cr(VI) concentration (calibration curve) is reported in Figure 5, and a very good fit with linear behavior can be found in the range 1–100 µM (squares). Remarkably, a linear behavior was also observed in the difference between the absorbance at 550 nm and that at 700 nm, herein referred to as ΔA_550/700_ (stars). The measurement of this quantity makes the method less affected by spurious variations of transparency and could enable real colorimetric detection.

It should be pointed out that, differently from the great majority of colorimetric methods (see, for instance, [11,12,13,16,29,30]), the interception of the calibration curve with the y-axis is close to zero, even for the absolute measurement at 550 nm. This is due to the fact that the absorbance of the reference solution, i.e., with a null concentration of Cr(VI), is lower than 2 × 10^−3^ OD in the range between 550 and 700 nm. Such a low value is below the photometric accuracy of the spectrophotometry used in this study (4 × 10^−3^ OD), and the absorbance variations due to the presence of Cr(VI) were substantially detected against the instrumental background. For this reason, the signal-to-noise ratio of the experiment was estimated based on the standard deviation σ of the lowest concentration spectrum. Specifically, the LOD was determined according to the formula LOD = 3σ/S, where S is the sensitivity, i.e., the slope of the calibration curve. The values of LOD = 300 nM and LOD = 600 nM were found based on the absorbance measurement at 550 nm and the ratiometric measurement of ΔA_550/700_, respectively.

#### 3.2.1. Selectivity for Cr(VI) Ions

**Figure 6:**
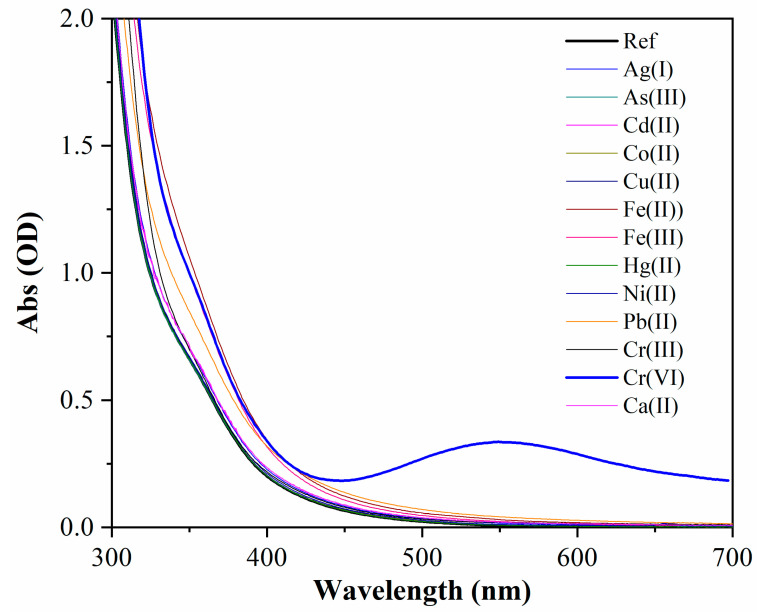
Selective response of UV-vis absorption spectra of N-CDs-W-THF upon the addition of different HM ions at a concentration of 100 µM and calcium ions at 1 mM. Note that As(III) is present as the anion AsO_3_^3−^ in water, which is in contrast to all the other metals, which are present as cations.

The selectivity of N-CDs-W-THF was tested over the most common heavy metals using the same assay procedure as the one used for Cr(VI). Figure 6 shows the UV-vis absorption spectra of the sensing solution after the addition of sample water solutions with different metal ions, including Cr(VI), at a concentration of 100 µM. Calcium at a concentration of 1 mM was also considered, as it is a common major component of the permanent hardness of many drinking waters.

**Figure 7:**
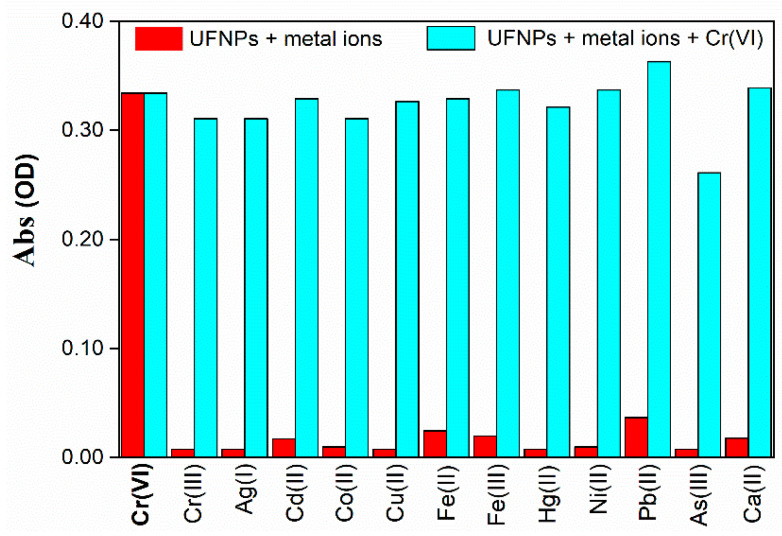
Absorbance of N-CDs-W-THF solution at 550 nm upon the addition of different HM ions in the absence (red bars) and in the presence (cyan bars) of Cr(VI) at a concentration of 100 µM. Calcium ions at a concentration of 1000 µM were also added for comparison. Note that As(III) is present as the anion AsO_3_^3−^ in water, which is in contrast to all the other metals, which are present as cations.

A strong selectivity was demonstrated in that the arising of the characteristic absorption band at 550 nm was only observed in the presence of Cr(VI), while the other ions did not significantly alter the transparency in the visible range and only produced minor absorbance variations on the UV side of the spectrum. In particular, N-CDs-W-THF clearly distinguished between Cr(III) and Cr(VI). These results are summarized in the diagram in Figure 7, where the absorbance at 550 nm is reported for the different ionic species (red bars). In addition, Figure S5 shows that the pH conditions of the water samples had little effect on the response to Cr(VI) in terms of values in the range from 3 to 11. In fact, the absorption band only experienced a small blue shift and an increase of absorbance on the short-wavelength side were observed at low pH values. This is also due to the optimized 1:2 ratio, which effectively diluted the sample solution.

#### 3.2.2. Interfering Effects from Other HMs

The possible influence on the response to hexavalent chromium, coming from the presence of other HM anions, was tested by performing the sensitivity experiment with a solution containing both Cr(VI) and another metal ion, with each species at a concentration of 100 µM. As displayed in the absorption spectra of Figure S6 and represented in the diagram of Figure 7 (cyan bars), only arsenic showed an appreciable masking effect on the detection of Cr(VI), with a percentage variation of response close to −20%. However, this low decrease of sensitivity is even less significant considering that arsenic is a highly toxic species, with a threshold limit that is five times lower than that of Cr(VI) and is expected to be present in much smaller quantities in the majority of water samples.

#### 3.2.3. Practical Application to Real Samples

The practical applicability of this method was investigated by performing a standard sensing experiment on spiked samples of real tap water collected from the city water main of Rome (South-East area of the city) and from lake water coming from Lago Albano, a volcanic lake 20 km south-east of Rome. Specifically, 1 mL of a real water sample was spiked with 10 μL of Cr(VI) standard solution at appropriate concentrations, and the sample was then added to 2 mL of N-CDs-W-THF sensing solution without any previous treatment such as filtering, centrifugation, acid digestion, or boiling. The respective absorption spectra are reported in Figure S7, while Table 2 shows that the percentage recovery of the spiked samples for the lake water was in the range from 98% to 110%, which represents a good result, especially when considering that the assay did not undergo any pretreatment. In fact, Figure S8 shows that the calibration curve obtained in the DI water is essentially reproduced. In the tap water samples, recovery varied from 95% to 107% in the high concentration range and worsened to 121% for the lowest measured value of 1 µM. Taking into account the high permanent hardness of the drinking water in Rome (33°fH), with a calcium content as high as 101 mg/L (2.5 mM), these results are also reasonably compatible with the real practicability of this method.

#### 3.2.4. Comparison with Other Sensing Materials and Techniques

In Table 3, the detection limit and linearity range of the present method are compared to those of other nanomaterials that have recently been reported in terms of colorimetric detection. Examples of the dye-based colorimetric method and fluorometric and electrochemical detection are also reported for comparison. It is clear that the LOD of the present system (0.3 µM) is outperformed by that of other nanomaterial systems [11,13,31,32,33], but it is still lower than the guideline value set by the WHO (0.962 µM). On the other hand, the linearity range is the broadest of all of the other colorimetric-based methods and is only paralleled by fluorescence quenching methods [30,34,35], dye-based colorimetry [9], and electrochemistry [36], which achieved similarly high values of LOD. In particular, in the present system the linear calibration curve showed a zero-intercept, which reflects the negligeable absorbance of the sensing material. In addition, it should be noted that most of the nanomaterials reported in the literature are based on gold or silver nanoparticles [11,12,13,33], which can be affected by tricky and non-reproducible sysnthesis methods and by critical stability over time. More importantly, all of the listed methods are based on reactant solutions that must be prepared just before the measurements are taken and require quite complex sensing procedures. For instance, the detection of chromium with Poly (N-Phenylglycine) nanoparticles (PNPG-PEG) [29] required mixing Cr(VI) sample solution with 37.5 μL of PNPG-PEG (1 mg/mL), 112.5 μL of hydrogen peroxide (100 nM), and 300 μL of 3,3,5,5-tetramethylbenzidine (10 mM) in acetate buffer (pH 4). The mixture had to be kept at 35 °C for 30 min. The use of Au nanorods [11] involved the addition of 0.5 M HCl to maintain the acidic condition and then needed to undergo incubation at 50 °C for 30 min. Even simpler methods, such as the ones using carbon dots, needed Tris-HCl buffer [35] or phosphate-buffered saline solution [30] to maintain pH = 7.4. Differently, the N-CDs-W-THF solutions used in the present method demonstrated 9-month stability and thus could be prepared in advance and stored for future assays. The sensing experiment only requires the addition of the water sample to the sensing solution, and the results can be obtained in 1 min at room temperature without the needs for any further procedures.

#### 3.2.5. Possible Mechanism for the Interaction of Cr(VI) with N-CDs-W-THF

A possible, suitable hypothesis for the mechanism of absorbance variations relates to the formation of Cr(VI) complexes with the functional groups of N-CDs-W-THF with higher affinity with respect to the other HMs. This peculiar interaction is determined by the combination and exposure of the functional groups on the surface of the CDs and the formation of hydrogen bonds with THF. The latter, in particular, varies the electronic density on the CDs and is likely responsible for the peculiar absorption at 550 nm since it prevents the ligand–metal charge back-donation, thus decreasing the t_2g_e_g_* Cr(VI) gap.

To this regard, a sensing response due to the inner filter effect (IFE) can be ruled out since the absorption spectrum of N-CDs-W-THF after interaction with Cr(VI) is quite different from the bare addition of the respective spectra of the two species (Figure S4), which is in contrast with what was reported in the water solutions of N-doped CDs prepared by bottom-up synthesis [37]. In fact, to the best of our knowledge, this is the first case of a colorimetric response to chromium, i.e., through the variation of optical absorbance, observed in CDs, which are usually used as fluorimetric materials [30,34,35,38,39].

The colorimetric detection by ligand affinity has advantages over assays based, for instance, on the peroxidase mimicking of TMB (tetramethylbenzidine) oxidation by H_2_O_2_ [29,40] due to the more stable set-up. The employment of H_2_O_2_, in fact, may introduce response fluctuation due to its instability.

## 4. Conclusions

In summary, through the one-pot oxidative cage opening of C_60_ fullerene in basic ambient, we obtained stable water solutions of N-doped carbon dots that had subsequently had THF added. The resulting N-CDs-W-THF have a smaller hydrodynamic range compared to N-CDs-W, which is likely due to the strong hydrogen bonding, as determined by IR. Possibly as a consequence of hydrogen bonding, the N-CDs-W-THF showed a peculiar selective colorimetric response to the presence of Cr(VI) ions in water that had never been observed in carbon dots. The variation in the optical absorbance could be observed in the visible spectral range against the null optical baseline of the sensing solution and was linear in the broad interval of 1–100 µM. This enabled a limit of detection of 300 nM, which is higher than that obtained with other nanomaterials but below the guideline value set by the WHO (962 nM). Moreover, linear behavior was also observed during ratiometric determination at two different wavelengths, which could reduce the influence of interferents and transparency variants due to other factors. More importantly, and differently from other systems, the reagent solutions were stable for at least 9 months, and the sensing procedure was extremely simple, cost-effective, and fast. The optimized method only required the addition of the water sample to the sensing solution in a 1:2 ratio and a reaction time of 1 min. Moreover, it showed low sensitivity to the pH of the water samples, with the pH being in in the range of 3 to 11. We believe that these characteristics make the optical detection of Cr(VI)) trough N-CDs-W-THF a promising method for the user-friendly, onsite detection of this dangerous water contaminant. To this end, further studies are in progress to deposit and/or incorporate N-CDs-W-THF into the solid transparent matrices for more practical sensing systems.

## Figures and Tables

**Table 1:** Average hydrodynamic diameter 2RH from DLS measurements determined by intensity-weighted size distribution NNLS algorithm.

Sample	Addition	Diameter (nm)	Error (nm)
N-CDs-W		3.50	±0.20
N-CDs-W-THF		3.12	±0.30
N-CDs-W-THF	Cr (IV) 5 mM	4.00	±0.09

**Table 2:** The recovery of Cr(VI) ions in real lake and tap water samples.

Sample	Spiked Cr(VI) (µM)	Found Cr(VI) (µM)	Recovery (%)
Lake water Lago Albano (Italy)	50	53	106
	10	9.8	98
	5	5.4	108
	2	2.2	110
	1	1.1	110
	50	47	95
Tap water	10	10.7	107
(Rome, Italy)	5	5.2	104
	2	2.2	110
	1	1.21	121

**Table 3:** Comparison of the performances of various techniques and materials for Cr(VI).

Materials	Method	Linear Range (µM)	Limit of Detection (µM)	Reference
Poly (N-Phenylglycine) NPs	Colorimetric	0.01–0.1	0.012	[29]
“ ”	Colorimetric	0.05–12.5	0.52	[29]
Cu-decorated GO	Colorimetric	0–0.2	0.067	[31]
Au NPs	Colorimetric	0.5–50	0.280	[32]
Au nanorods	Colorimetric	0.1–20	0.088	[11]
PVP-Ag NPs	Colorimetric	0.1–2.4	0.034	[13]
Au NPs	Colorimetric	0.15–2	0.1	[33]
citrate-capped Au NPs	Colorimetric	2–10	0.3	[12]
GO NPs	Colorimetric	0.07–0.43	0.0058	[16]
Carbon dots	Fluorescence	0.5–260	0.260	[30]
Carbon dots	Fluorescence	1–400 *	0.13	[34]
Carbon dots	Fluorescence	1–100	0.25	[35]
1,5-Diphenylcarbazide	Colorimetric	0.6–140	0.46	[9]
Au decorated/CNTs	Electrochemistry	0.8–230	0.72	[36]
N-CDs-W-THF	Colorimetric	0–100	0.30	This study

* on a semi-log scale.

## Supplementary Materials

The following are available online at https://www.mdpi.com/article/10.3390/s21196353/s1, Figure S1: X-ray powder diffraction pattern of synthesized N-CDs, Table S1: Main IR peaks of the pristine fullerene-C_60_, water–THF mixture (W-THF) N-CDs-W, N-CDs-W-THF, and corresponding assignments, Figure S2: Dependence on the excitation wavelength of fluorescent emission intensity and peak wavelength in N-CDs-W-THF, Figure S3: White-light image of the pristine N-CDs-W-THF sensing solution (Ref) and after the addition of different HM ions at a concentration of 100 µM and calcium ions at 1 mM, Figure S4: UV-vis absorption spectra of N-CDs-W-THF solution (black curve), Cr(VI) in DI water at 100 μM (blue), N-CDs-W-THF solution (red), and N-CDs-W solution (green) in the presence of Cr(VI) ions at a concentration of 100 µM, Figure S5: (a) UV-vis absorption spectra of N-CDs-W-THF and (b) absorbance of the sensing solution at 550 nm upon the addition of DI water with 100 μM of Cr(VI) ions at different pH values in the optimized ratio 2:1, Figure S6: UV-vis absorption spectra of N-CDs-W-THF reference solution upon the addition of Cr(VI) and other interfering HM ions at a concentration of 100 μM and calcium ions at 1 mM, Figure S7: UV-vis absorption spectra of N-CDs-W-THF reference solution upon the addition of (a) tap water and (b) lake water spiked with different concentrations of Cr(VI), Figure S8: Absorbance at 550 nm as a function of Cr(VI) concentration in tap water samples (circles) and lake water samples (stars). The dotted line is the calibration curve obtained with DI water.
