# Supplementary material for: Colorimetric Detection of Chromium(VI) Ions in Water Using Unfolded-Fullerene Carbon Nanoparticles
Source: Sensors (Basel). 2021 Sep 23;21(19):6353. doi: 10.3390/s21196353 (PMC8512488; doi:10.3390/s21196353)
Supplement: Supplementary file 1 [file sensors-21-06353-s001.zip › sensors-1371803-supplementary.pdf]

Supplementary Materials

Colorimetric Detection of Chromium(VI) Ions in Water Using Unfolded-Fullerene Carbon Nanoparticles

Saeedeh Babazadeh [1,2], Ramanand Bisauriya [1], Marilena Carbone [3,*], Ludovica Roselli [3], Daniele Cecchetti [3], Elvira Maria Bauer [4], Simona Sennato [5], Paolo Prosposito [1] and Roberto Pizzoferrato [1,*]

1 Department of Industrial Engineering, University of Rome Tor Vergata, 00133 Rome, Italy; s.babazadeh1990@gmail.com (S.B.); r.bisauriya@gmail.com (R.B.); paolo.prosposito@uniroma2.it (P.P.)

2 Department of Mechanical Engineering of Biosystems, Agriculture Faculty, Urmia University, 5756151818 Urmia, Iran

3 Department of Chemical Science and Technologies, University of Rome Tor Vergata, 00133 Rome, Italy; ludovica.roselli@alumni.uniroma2.eu (L.R.); daniele.cecchetti@uniroma2.it (D.C.)

4 Institute of Structure of Matter (ISM), Italian National Research Council (CNR), 00015 Rome, Italy; elvira.bauer@ism.cnr.it

5 Institute for Complex Systems (ISC), Italian National Research Council (CNR) and Physics Department, Sapienza University of Rome, 00185 Rome, Italy; simona.sennato@roma1.infn.it

* Correspondence: carbone@uniroma2.it (M.C.); pizzoferrato@uniroma2.it (R.P.)

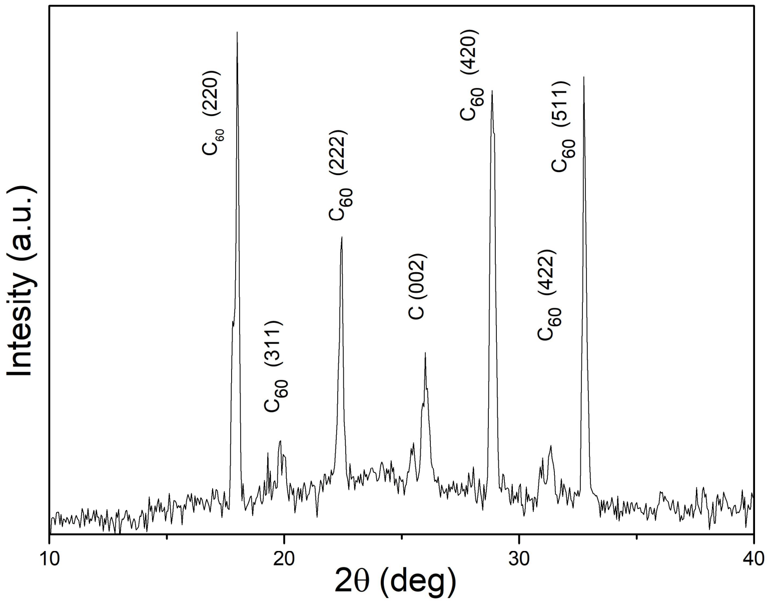

Figure S1. X-ray powder diffraction pattern of synthesized N-CDs.

Table S1. Main IR peaks of the pristine fullerene-C_{60}, mixture water-THF (W-THF) N-CDs-W, N-CDs-W-THF and corresponding assignments; s=strong, m=medium, w=weak, br=broad, sp=sharp, sh=shoulder.

**Fullerene**	**W-THF**	**N-CDs-W**	**N-CDs-W-THF**	**Assignments**
		3430 m br	3390 m br	-O-H stretching
		3240 m br	3205m br	-N-H asymmetric stretching
	3360 br			-O-H stretching
		3192 m sh		-N-H symmetric stretching
		3047 m br	3053 m br	-CH aromatic stretching
			2953 m br	-CH_x stretching
	2974 w			-CH_2 asymmetric stretching
	2877 w			-CH_2 symmetric stretching
		2866 sh	2882 w br	-CH_x stretching
		1753 vw		-C=O carboxylic stretching
			1707 s sp	-C=O carboxylic stretching
		1672 vw br		-C=O primary amide stretching
	1635m			-O-H bending
		1630 m sp		-C=C, -C=O stretching, -NH_2 scissoring
		1593 vw br		-C=C, -C=O stretching, -NH_2 scissoring
			1548w br	-C=C, -C=O stretching, -NH_2 scissoring
	1465 w			-CH_2 deformation bending
1427 m sp				-C=C- tangential displacement
		1409 s br	1406 s br	-C-N amide III stretching, -C-O hydroxyl stretching
			1329 s br	-C-OH carboxylic acid stretching
	1367 w			-CH_2 deformation bending
		1307 s br		-C-OH carboxylic acid stretching
			1245 m br	-CH_x wagging
			1209 m sh	-CH_x wagging
			1178 m sh	-C-OH carboxyl stretching
	1190 w			O-H···O-C stretching
1180 m sp				-C=C- tangential displacement
		1094 w sh		-C-O-C asymmetric ether stretching
	1055 br		1055 s br	–C-O-C- asymmetric stretching
		1042 m sp	1032 m sh	-C-O-C asymmetric ether stretching
			1022 m sh	-C-O-C asymmetric ether stretching
	914 w			-CH_2-CH_2- stretching
			935 w sp	-NH wagging, -C-O-C symmetric ether stretching
			905 w sp	-C-O-C symmetric ether stretching,
			874 vw br	-NH wagging, -C-O-C symmetric ether stretching
		827 m sp	826 s sp	-C-O-C epoxy bending
	807 w			
		714 m sp		-NH_2 wagging
572 s sp				-C-H radial displacement
522 s sp				-C-H radial displacement

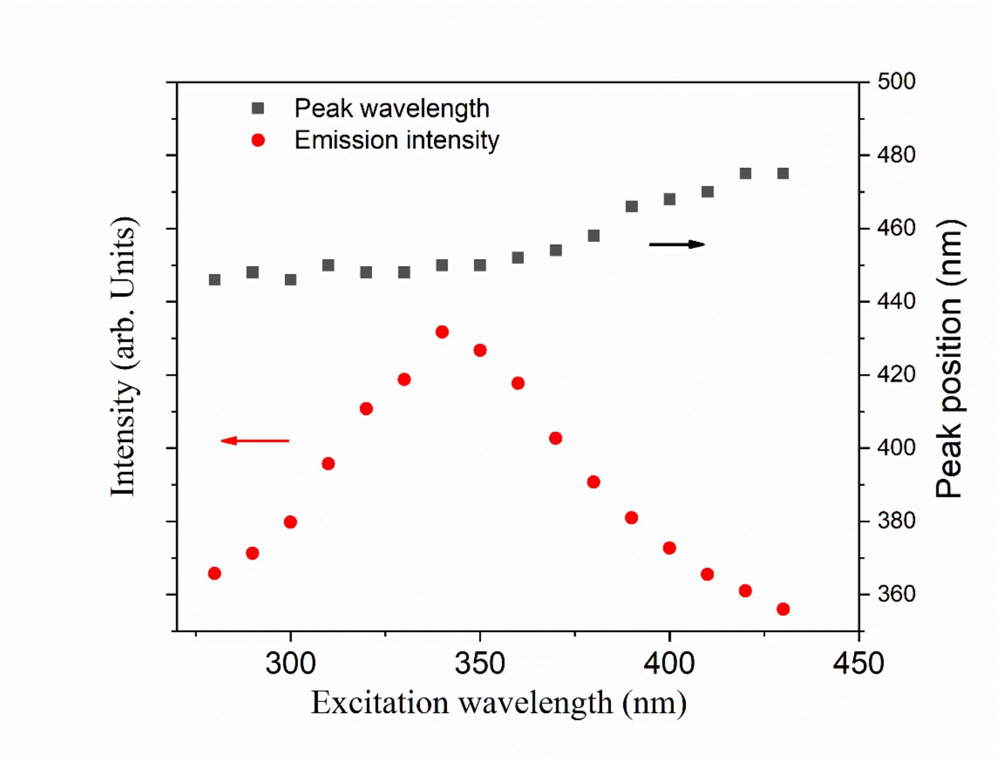

Figure S2. Dependence on the excitation wavelength of fluorescent emission intensity and peak wavelength in N-CDs-W-THF.

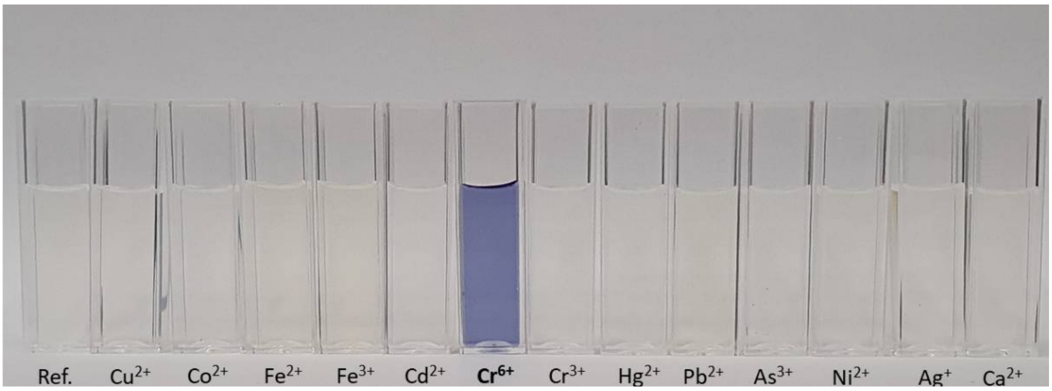

Figure S3. White-light image of the pristine N-CDs-W-THF sensing solution (Ref) and after the addition of different HM ions at a concentration of 100 µM and calcium ions at 1 mM.

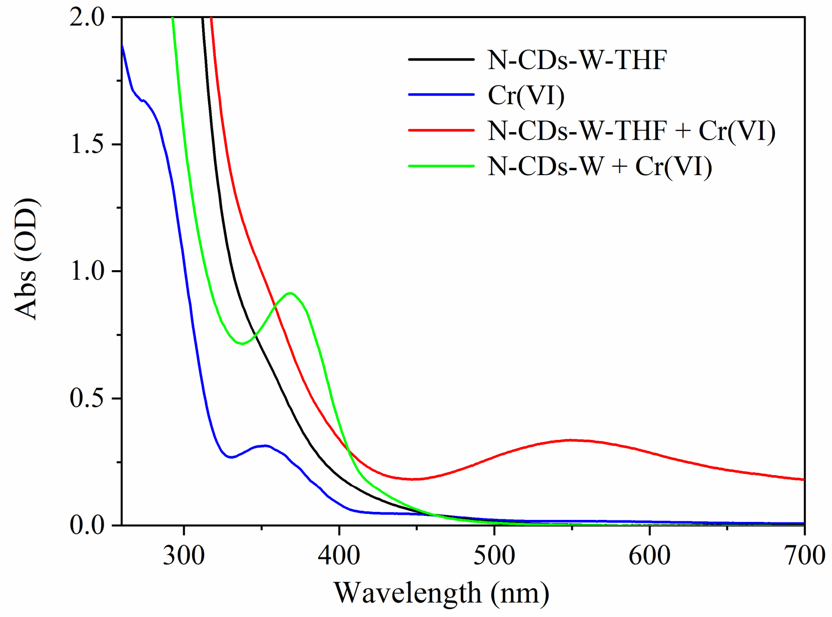

Figure S4. UV-vis absorption spectra of N-CDs-W-THF reference solution (black curve), Cr(VI) in DI water at 100 μM (blue), N-CDs-W-THF solution (red) and N-CDs-W solution (green) in the presence of Cr(VI) ions at a concentration of 100 μM.

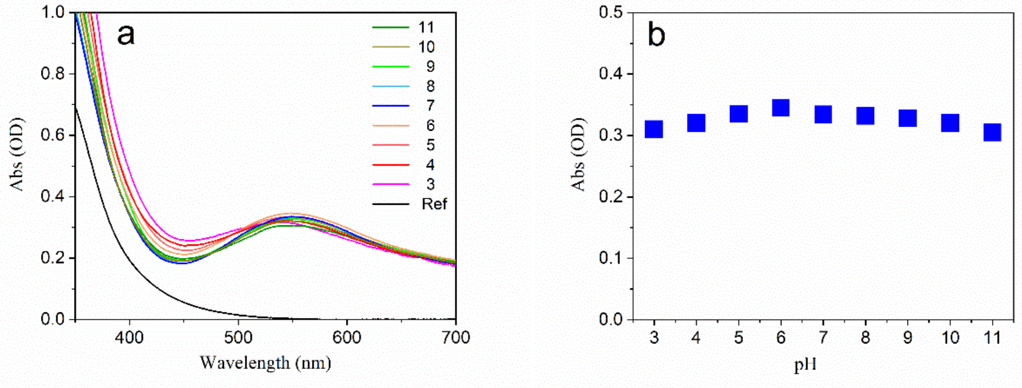

Figure S5. (**a**) UV-vis absorption spectra of N-CDs-W-THF and (**b**) absorbance at 550 nm of the sensing solution upon the addition of DI water with 100 μM of Cr(VI) ions at different values of pH in the optimized volume ratio 2:1.

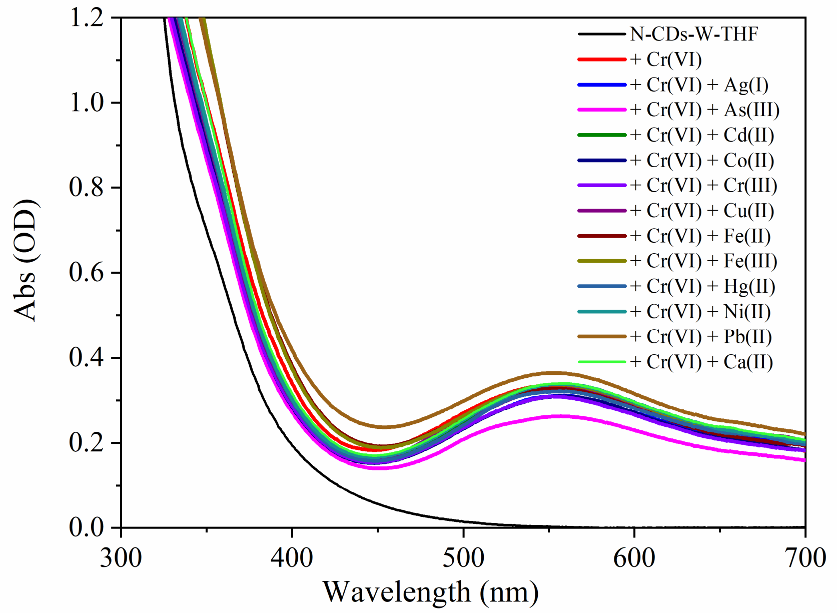

Figure S6. UV-vis absorption spectra of N-CDs-W-THF reference solution upon the addition of Cr(VI) and other interfering HM ions at a concentration of 100 μM and calcium ions at 1 mM.

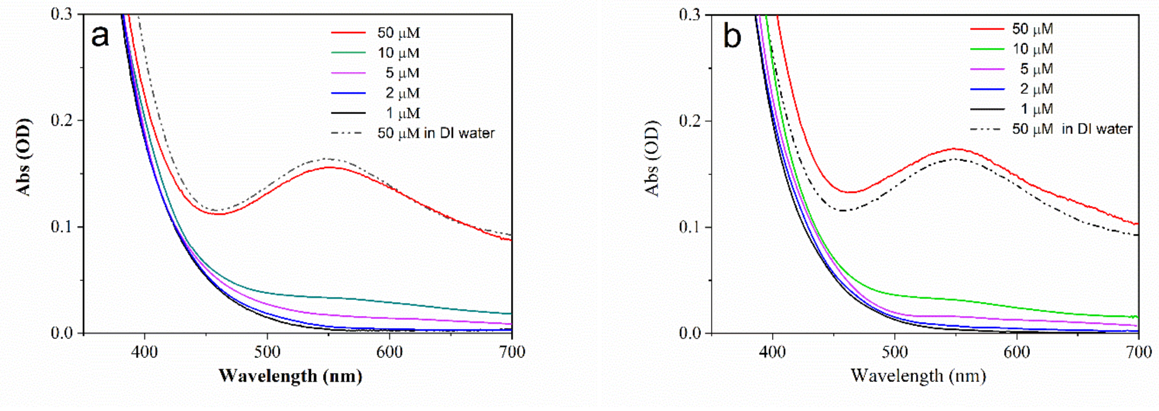

Figure S7. UV-vis absorption spectra of N-CDs-W-THF reference solution upon the addition of (**a**) tap water and (**b**) lake water spiked with different concentrations of Cr(VI).

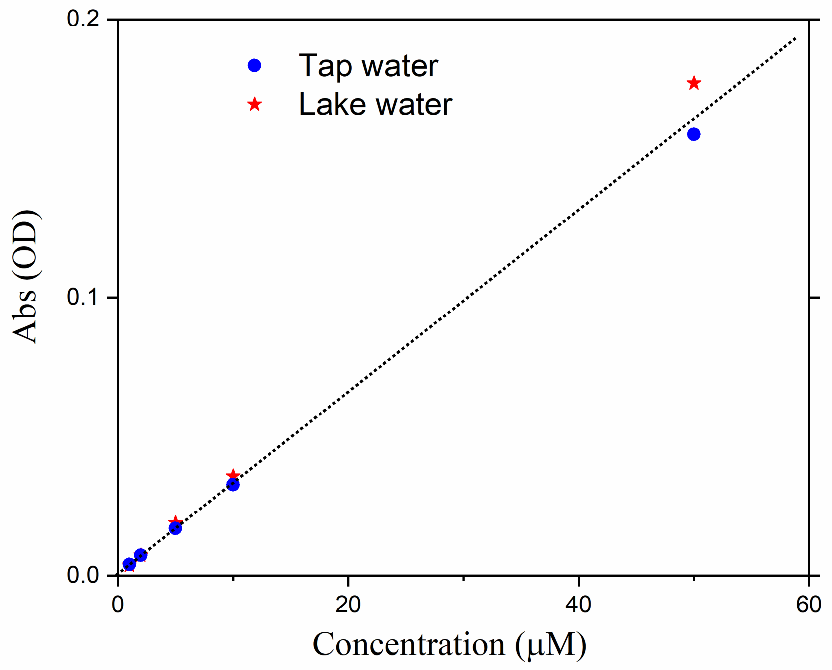

Figure S8. Absorbance at 550 nm as a function of Cr(VI) concentration in tap water samples (circles) and lake water samples (stars). The dotted line is the calibration curve obtained with DI water.
